# Side-by-Side Comparison of the In Vivo Performance of [^212^Pb]Pb-DOTAMTATE and Other SSTR2-Targeting Compounds

**DOI:** 10.2967/jnumed.124.268345

**Published:** 2025-03

**Authors:** Amal Saidi, Tania A. Stallons, Amy G. Wong, Aaron T. Schatzmann, Ugur Soysal, Julien J. Torgue

**Affiliations:** 1 Orano Med SAS, Paris, France; and; 2 Orano Med LLC, Plano, Texas

**Keywords:** [^212^Pb]Pb-DOTAMTATE, PRRT, SSTR2 analogs, targeted α-therapy

## Abstract

There are numerous versions of octreotide and octreotate, including DOTAMTATE, DOTATATE, JR11, and lead-specific chelator (PSC)-PEG2-TOC. These peptides, which can be either analogs or antagonists, are used in nuclear medicine for diagnostic imaging or targeted radionuclide therapy of neuroendocrine tumors that are positive for somatostatin receptors (SSTRs). Despite their structural and targeting similarities, they have distinct properties and clinical uses. We aimed to perform an extensive preclinical comparison of all these somatostatin analogs with ^212^Pb, directly studying their pharmacokinetic properties in tumors overexpressing SSTR2. **Methods:** All SSTR2 analogs were manufactured with the DOTAM, PSC, or DOTA chelators for appropriate comparison after radiolabeling with ^212^Pb. Chelation, quantification, and pharmacokinetics were compared side by side in AR42J-tumor–bearing animals. **Results:** These findings highlight the superior chelation efficiency and faster kinetics of DOTAM and then DOTA compared with the PSC. We also discovered a superior tumor-to-kidney area under the curve ratio for [^212^Pb]Pb-DOTAMTATE over other SSTR2-targeting peptides when radiolabeled with ^212^Pb. **Conclusion:** Taken together, the results indicates that [^212^Pb]Pb-DOTAMTATE has favorable tumor retention and a more favorable dosimetry profile, which is crucial for targeted α-therapy in treating SSTR2-positive neuroendocrine tumors.

## Visual Abstract

**Figure:**
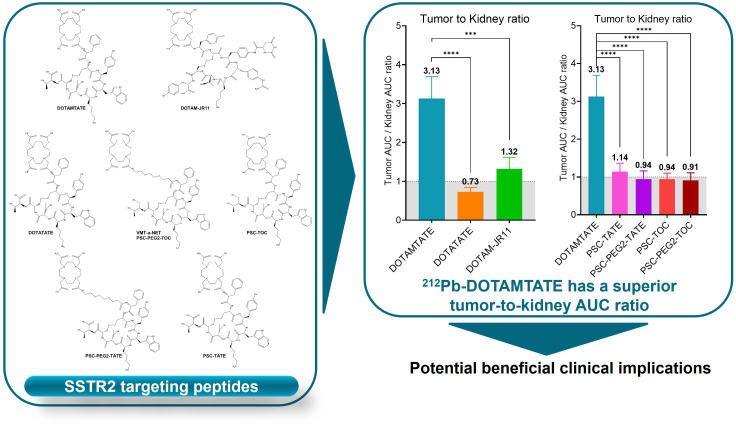

Somatostatin receptor 2 (SSTR2) has significant relevance because of its overexpression in certain types of neuroendocrine tumors (NETs). NETs are a heterogeneous group of tumors that arise from neuroendocrine cells present throughout the body, most commonly in the gastrointestinal tract, pancreas, and lungs. These tumors can exhibit varying degrees of aggressiveness and hormone secretion (1). In recent years, peptide receptor radionuclide therapy has emerged as an effective treatment option for patients with SSTR2-positive NETs. Peptide receptor radionuclide therapy combines somatostatin analogs with a therapeutic radionuclide, such as ^177^Lu, ^90^Y, or ^212^Pb, which emits radiation to selectively target and destroy SSTR2-expressing tumor cells. This approach has revolutionized the management of NET cancers, providing improved treatment outcomes and better quality of life for patients (2,3).

^212^Pb is the parent of ^212^Bi, an α-emitting radionuclide with a short half-life, meaning it emits an α-particle during its decay process. α-particles have a short range in tissues, allowing for highly localized delivery of radiation and the potential to induce DNA damage and cell death in tumor cells while sparing surrounding healthy tissues (4). This characteristic makes ^212^Pb an attractive option for targeted α-therapy.

[^212^Pb]Pb-DOTAMTATE, also known as AlphaMedix, is a radiotherapeutic compound that combines the targeting capabilities of the somatostatin analog DOTAMTATE with the therapeutic properties of the radioactive isotope ^212^Pb. Preclinical and early clinical studies have shown promising results for [^212^Pb]Pb-DOTAMTATE in the treatment of SSTR2-positive NETs (5–7). However, to the best of our knowledge, it has never been compared with the other somatostatin analogs or antagonists in the same preclinical setting, coupled with ^212^Pb. DOTATATE, JR11 (SSTR2 antagonist), and lead-specific chelator (PSC)-diethylene glycol (PEG2)-TOC are known somatostatin analogs used in nuclear medicine for diagnostic imaging or targeted radionuclide therapy of somatostatin receptor–positive NETs. Although they share similarities in their structure and targeting capabilities, there are differences in their specific properties and clinical applications.

The 2 most common SSTR2 ligands are based on octreotate or octreotide. Whereas both TATE and TOC target SSTR2, TATE binding to SSTR2 is approximately 10-fold higher than that of TOC (8). Also, TOC exhibits broader binding affinity for additional receptor subtypes as it presents high affinity not only for SSTR2 but also for somatostatin receptor 3 and somatostatin receptor 5. TATE has a moderate affinity for somatostatin receptor 5 and only minimal binding to somatostatin receptor 3 (9). TATE has been specifically designed to target SSTR2.

JR11 is one of the SSTR2 antagonists (10). The observation that G protein–coupled receptor antagonists may bind to more binding sites than agonists, since their binding is independent of the fraction of receptors coupled to the guanosine triphosphate–binding proteins (11), was the primary reason for the development of radiolabeled SSTR2 antagonists. Structurally, the main feature to convert an agonist to an antagonist was shown to be the inversion of chirality at positions 1 and 2 of the octreotide family (12).

All bifunctional chelators used in radiotherapy applications form conjugates with a vector of interest to allow for the delivery of the isotope payload to the target site. The choice of a particular bifunctional chelator will depend on the conjugation strategy (*N*-hydroxysuccinimide, isothiocyanate, cysteine labeling, or click chemistry, to mention some), as well as the isotope chosen to diagnose or treat the indication in question. Among these bifunctional chelators, DOTA (also known as tetraxetan), DOTAM (also known as TCMC), and PSC are 3 examples of macrocyclic chelators that are structures based on the cyclic polyamine tetraazacyclododecane, also known as cyclen. DOTA is widely used in nuclear medicine applications and is an excellent chelator of ^177^Lu for therapy and of ^68^Ga for diagnostic imaging. However, it is suboptimal for ^212^Pb chelation (13). This deficiency was addressed with the development of DOTAM. The substitution of the carboxylate arms for amides in the original DOTA structure resulted in better chelation kinetics, as well as better thermodynamic stability when complexed with lead (14). As the field of nuclear medicine has taken center stage in the medical field, with the promise of better treatment outcomes and lower side effects for the patient, the research community has turned its attention to studying, among other things, the mechanisms of excretion by the body, particularly the renal pathway, in which kidneys are the organs that receive the highest dose of radiation in the body during drug clearance. Among the variables under study, many sustain that the overall charge of the lead complex core in the payload is among the most relevant of them, because of the possible electrostatic interactions that may take place with the highly anionic nature of the kidneys. Hence, some groups have taken on the task of designing chelators that can have different charges on complexation with the lead atom. One such example is the ligand PSC. When compared with Pb-DOTA (charge of −2) and DOTAM (charge of +2), PSC can form neutral complexes with lead, because of the combination of amide and carboxylic acid arms present in the ligand. These differences in functional groups and charge can influence their chelating properties, applications, and the molecules’ pharmacokinetics.

Finally, analogs can differ in their linkers. DOTATATE, DOTAMTATE, and DOTAM-JR11 do not have a linker between the ligand and the chelator, but PSC-PEG2-TOC has a PEG2 linker. Polyethylene glycol (PEG) linkers are chemically functionalized. PEG linkers are particularly interesting because of their aqueous solubility and nonimmunogenicity. Their use can impact the pharmacokinetics of molecules.

In this study, our objective was to conduct a comprehensive preclinical comparison of all these somatostatin analogs with ^212^Pb, examining their pharmacokinetic properties in a side-by-side and direct manner using SSTR2-overexpressing tumors, AR42J.

## MATERIALS AND METHODS

### Cell Line and Mice

AR42J rat pancreatic cell line was purchased from American Type Culture Collection. The cells were tested for *Mycoplasma* by Hoechst DNA stain, agar culture, and polymerase chain reaction–based assay by American Type Culture Collection and were not detected as per certificate of analysis. The cells were maintained in F12K medium (Gibco) containing 20% fetal bovine serum (Gibco). Athymic nude and CD-1 mice were purchased from Envigo. All studies were conducted using female mice unless otherwise mentioned. All studies were conducted under the approval of the Institutional Animal Care and Use Committee and performed in compliance with guidelines from the Public Health Service Policy and Animal Welfare Act of the United States.

### Manufacturing

The PSC was prepared from DO2A-*tert*-butyl ester (Macrocyclics product M-120) in the following 3 main synthetic steps: (i) reaction of benzyl bromoacetate to the amine of M-120; (ii) reaction of bromoacetamide to the remaining amine of M-120; (iii) deprotection of the carboxybenzyl to afford the carboxylic acid for conjugation to the N-terminus of the peptide. The purity of the PSC was assessed using high-performance liquid chromatography, and its identity was confirmed by liquid chromatography–mass spectrometry.

Good manufacturing practice DOTAMTATE (C_65_H_93_N_17_O_16_S_2_) was purchased from Macrocyclics, Inc. DOTATATE was purchased from ABX (ABX Advanced Biochemical Compounds). DOTAM-JR11 (DOTAM-p-Cl-Phe-cyclo(d-Cys-Aph(Hor)-d-Aph(Cbm)-Lys-Thr-Cys)d-Tyr-ONH_2_, where p-Cl-Phe is *para*-chloro-l-phenylalanine, Aph(Hor) is 4-amino-l-hydroorotyl-phenylalanine, and d-Aph(Cbm) is 4-amino-d-Phe-carbamoyl), PSC-TATE (PSC-d-Phe-cyclo(Cys-Tyr-d-Trp-Lys-Thr-Cys)Thr-COOH), PSC-PEG2-TATE (PSC-amino-PEG2-acid-d-Phe-cyclo(Cys-Tyr-d-Trp-Lys-Thr-Cys)Thr-COOH), PSC-TOC (PSC-d-Phe-cyclo(Cys-Tyr-d-Trp-Lys-Thr-Cys)Thr-OH), and PSC-PEG2-TOC (PSC-amino-PEG2-acid-d-Phe-cyclo(Cys-Tyr-d-Trp-Lys-Thr-Cys)Thr-OH) (15) were each synthesized using a Biotage Alstra and initiator microwave peptide synthesizer with Fmoc solid-phase peptide synthesis. The JR11 peptide was synthesized on Chemmatrix Rink amide resin to afford an amidated C-terminus. The JR11 peptide was conjugated to DOTAM-monocarboxylic acid (Macrocylics product B-170) on the N-terminus while it was still on the resin. PSC-TATE and PSC-PEG2-TATE were synthesized on Fmoc-l-Thr(tbu)-Wang resin, whereas PSC-TOC and PSC-PEG2-TOC where synthesized on l-Thr-ol(tbu)-2-chlorotrityl chloride resin. PSC-TATE, PSC-PEG2-TATE, and PSC-PEG2-TOC were conjugated to the *tert*-butyl–protected version of PSC to the N-terminus of each resin-bound peptide. The peptide conjugates were cleaved from the resin and the protecting groups from PSC and the peptide side chains removed with a cocktail of trifluoroacetic acid and radical scavengers and then precipitated with diethyl ether. Each peptide was cyclized with 30% dimethyl sulfoxide in water. The crude was purified by reversed-phase liquid chromatography.

### Radiolabeling

The radioisotope-free compounds were diluted in metal-free water at needed concentrations and were stored at −20°C. ^212^Pb was produced using ^224^Ra generators (Orano Med LLC). Chelation was performed by incubating 27.5 nmol of peptide per 444 MBq of ^212^Pb for 10 min at room temperature in 0.4 M ammonium acetate buffer, with a pH of 6.1. Radiochemical purity, assayed by instant thin-layer chromatography was used to confirm that ^212^Pb chelation was greater than 95%. Radiolabeled peptides were spotted onto an instant thin-layer chromatography strip and allowed to migrate in a 22% citrate buffer. The instant thin-layer chromatography strip was then cut in half and counted using a calibrated Perkin Elmer Wizard2 2470 automatic γ-counter with an energy window of 185–250 keV considering ^212^Pb major emitted γ-rays (238 keV). Samples were diluted to appropriate activity before injection in a buffer containing ascorbic acid, ethanol, and polysorbate-20.

### Measurement of ^212^Pb Radioactivity

A high-purity germanium detector was calibrated using a 1 μCi National Institute of Standards and Technology (NIST) ^152^Eu source (0117182) and a 1 μCi NIST ^133^Ba source (0117181) to accurately quantify ^212^Pb at the distinguished 238 keV γ-ray peak. Samples of 5 μL of ^212^Pb in solution were spotted onto Whatman filter paper (Millipore-Sigma) and measured on the high-purity germanium, resulting in accurate concentration measurements. For instrument comparison purposes, a Capintec CRC-55TR dose calibrator and a Biodex Atom Lab 500 Plus dose calibrator were used to measure ^212^Pb at various dial values and calibration numbers. Biodex refers to dial value as a means of calibrating the source activity measured for an isotope because of the differences in detector current. This is equivalent to what Capintec calls the calibration number. The dial values for both dose calibrators were confirmed with a ^212^Pb stock that had achieved equilibrium. A 5 μL sample of this stock was measured by the high-purity germanium and then extrapolated for total activity. This stock volume was placed into both dose calibrators, and the dial values were confirmed to match the total extrapolated measurement. These dial values were used for further comparison purposes.

### Tumor Models

For all tumor studies, 2 × 10^6^ AR42J cells were implanted subcutaneously, in an equal volume mixture of growth-factor-reduced Matrigel (Corning) and RPMI medium (Gibco), into the right flank of each mouse and grown to a volume of approximately 200–300 mm^3^.

### Biodistribution Studies

AR42J tumors were grown until an approximate tumor volume of 300 mm^3^ length, width, without height (150 mm^3^ length × width × height) was reached. In total, 100 μL of ^212^Pb SSTR2 analogs (∼0.37 MBq) were administered intravenously via the tail vein, and mice were euthanized at predetermined time points. The background was automatically subtracted from the counts. A standard was also used for decay correction to time of injection or time of collection. The percent injected dose per gram (%ID/g) was calculated for each organ collected.

### Radio–High-Performance Liquid Chromatography Studies

^212^Pb-labeled peptides were analyzed on an Agilent 1220 high-performance liquid chromatograph using a C18 reverse-phase column (Restek) with an acetonitrile gradient. Fractions were collected off the column every 6 s for a total of 8 min and then analyzed for radiometric detection with a Wizard 2470 automatic γ-counter (Perkin Elmer).

### Termination Criteria

Mice were sacrificed when tumor volumes reached 2,000 mm^3^ (length *×* width^2^) or 1,000 mm^3^ (length × width × height) or other predetermined termination criteria were met (weight loss >15% for 2 consecutive days or 20% weight loss from initial weight, serious bleeding, necrosis or ulceration of the tumor, scruffiness or lack of grooming over 5 d, lethargy over 3 d, weakness or balance issues over 5 d, hunchback appearance, diarrhea, or hypothermia).

### Statistical Analyses

Acquired data were statistically analyzed by a Student *t* test via GraphPad Prism software (version 10; Prism Inc). Acquired *P* values of less than 0.05 were considered statistically significant.

## RESULTS

### Comparison Between DOTAM, DOTA, and PSCs

**FIGURE 1.**
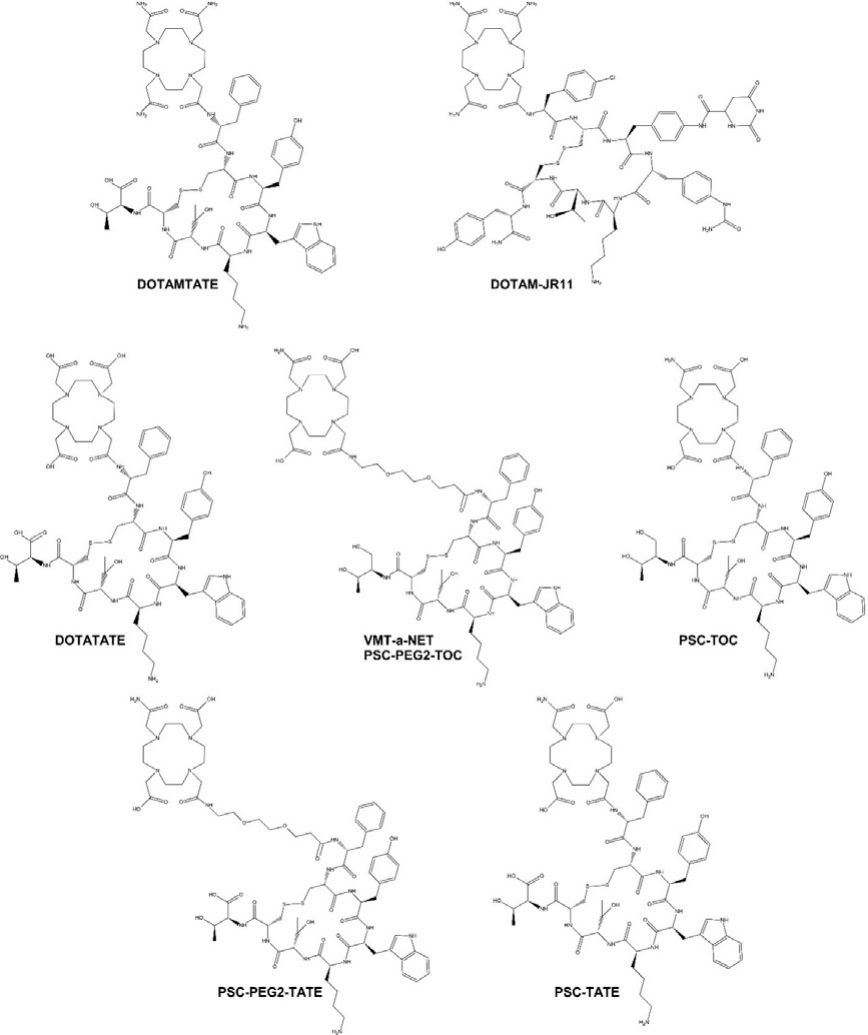
Chemical structures of SSTR2 analogs: DOTAMTATE (also known as AlphaMedix), DOTATATE, DOTAM-JR11, PSC-TATE, PSC-PEG2-TATE, PSC-TOC, and PSC-PEG2-TOC (also known as VMT-α-NET).

DOTAMTATE, DOTATATE, PSC-TATE, and PSC-PEG2-TATE (Fig. 1) were radiolabeled at a specific activity of 444 MBq per 50 μg (or 27.5 nmol) in a chelation buffer containing 5% ethanol, 20 mM ascorbic acid, and 0.02% polysorbate 80. The chelation of ^212^Pb appears to occur more slowly with the PSC than with the DOTA and DOTAM chelators. For DOTAM, an over 95% radiochemical yield (RCY) of ^212^Pb was achieved in less than 10 min at room temperature, whereas DOTA required an additional 20 min and PSC required a longer incubation of approximately 50 min, including 20 min at 37°C to reach an over 95% RCY (Table 1). The RCY remained above 95% at 24 h for all compounds.

**TABLE 1.** RCYs of Different Chelators Over Time*

Parameter	RCY of DOTAMTATE	RCY of PSC-TATE	RCY of PSC-PEG2-TATE	RCY of DOTATATE
10 min at RT	96.5% ± 0.7%	73.2% ± 6%	72.1% ± 4.7%	94.3% ± 0.7%
20 additional min at RT	—	93.3% ± 2.4%	89.9% ± 1.6%	99.2% ± 0.3%
20 additional min at 37°C	—	99.3% ± 0.3%	99.3% ± 0.1%	—
24 h at RT	99.0% ± 0.0%	98.7% ± 0.6%	98.7% ± 0.6%	98.3% ± 1.2%

* Values are RCYs for triplicate of each compound at each time point, expressed as a percentage ± SD. Please note that all measurements were conducted under identical conditions to ensure comparability, and all compounds were radiolabeled at a specific activity of 444 MBq/50 μg (or 27.5 nmol).

RT = room temperature.

### ^212^Pb Quantification

Some discrepancies in ^212^Pb quantifications were observed in various publications, so we sought to clarify the inconsistencies. In Li et al. (16), ^212^Pb was measured using a Capintec CRC25R dose calibrator and a dial setting of 158, according to the manufacturer’s instruction. The instruction provided a theoretic value for pure ^212^Pb without its daughters, which was different from the NIST recommendations for ^212^Pb quantification at the time (17). Therefore, we performed a side-by-side comparison using different dose calibrators from different vendors (Table 2). According to our analyses, we determined that a dial value of 688 should be the correct dial value for accurate ^212^Pb quantification when using the Capintec dose calibrator. This quantification is consistent with the most recent standards described by the NIST (18). The Capintec dose calibrator at a dial value of 158 shows 320% more activity than at a dial value of 688, leading to an approximate 3 times overestimation of the ^212^Pb, resulting in approximately 30 μCi per injected dose instead of the described 100 μCi administered in the toxicity study.

**TABLE 2.** Comparative Measurements of Dose Calibrators*

Dose calibrator	Dial value	^212^Pb measurement 6 h after elution (414.4 MBq of ^212^Pb)
Biodex Atom Lab 500Plus (model 086-336)	7.2	278.24 MBq at 3:03 PM
Capintec (model CRC-55TR)	688	277.87 MBq at 3:03 PM
Capintec (model CRC-55TR)	158	888 MBq at 3:03 PM

* Table presents measurements obtained from various dose calibrators. Each row represents unique dose calibrator, identified by its model and manufacturer. Columns represent dial values and measurement.

### SSTR2 Analog Drug Distribution Studies

**FIGURE 2.**
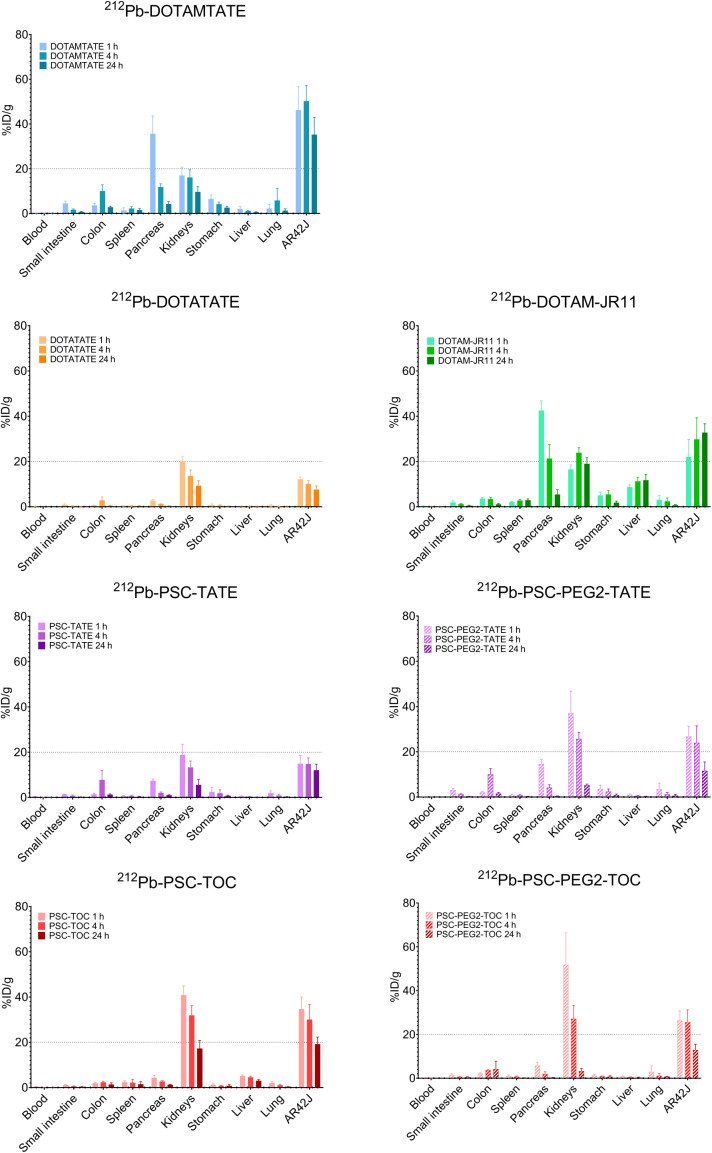
In vivo distribution of ^212^Pb-radiolabeled SSTR2 analogs in athymic nude female mice carrying subcutaneous AR42J tumors. Approximately 444 kBq per 27 pmol of drug was administered, and organs were collected from 4–5 mice per time point: 1, 4, and 24 h after injection. Tissue uptake is expressed as %ID/g ± SD decay corrected to time of injection (*n* = 4–5).

All studies were conducted in tumor-bearing female mice and showed that all SSTR2 analogs radiolabeled with ^212^Pb present a safe biodistribution profile with no unusual accumulation in healthy tissues (Fig. 2; Supplemental Figs. 1 and 2; supplemental materials are available at http://jnm.snmjournals.org). The pancreas and kidneys were the 2 healthy tissues with the highest drug uptake; however, the activity in the pancreas appeared higher for DOTAMTATE and DOTAM-JR11 but dropped rapidly between 4 and 24 h. Similarly, the uptake of [^212^Pb]Pb-DOTAMTATE and DOTAM-JR11 in healthy tissues such as the stomach, the lungs, and spleen was also observed to be higher compared with uptake of the other SSTR2 analogs. Meanwhile, the average tumor uptake 4 h after injection ranged from approximately 10 %ID/g for [^212^Pb]Pb-DOTATATE to approximately 50% for [^212^Pb]Pb-DOTAMTATE (Fig. 2).

### Area Under the Curve (AUC) Comparative Study

**FIGURE 3.**
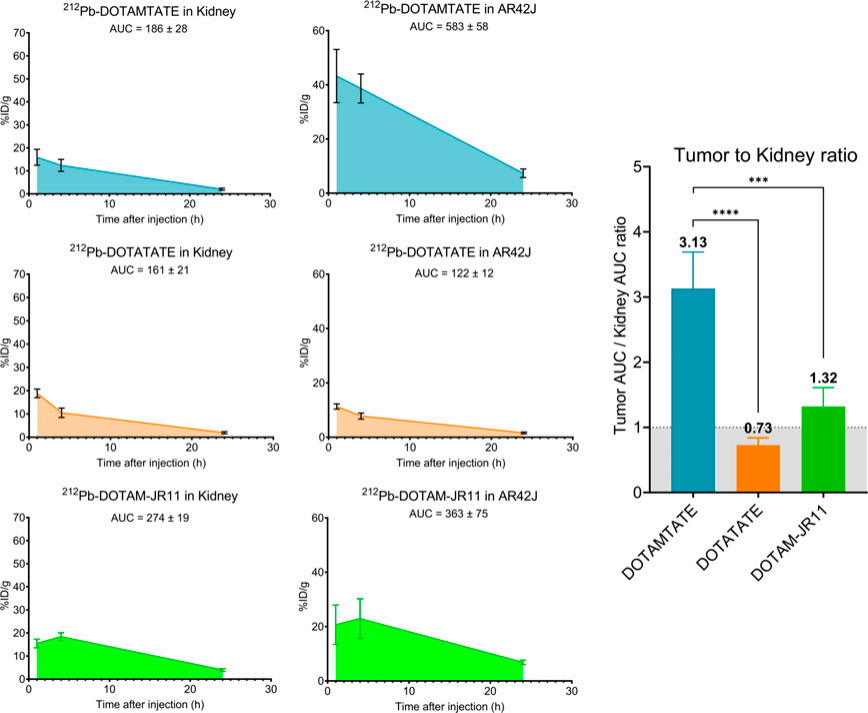
Tumor uptake AUC and ratios for ^212^Pb-radiolabeled SSTR2 analogs and antagonist. Between 370 and 444 kBq of drug was administered, and organs were collected from 4–5 mice per time point: 1, 4, and 24 h after injection. Tissue uptake is expressed as %ID/g ± SD decay corrected to time of collection (*n* = 4–5). ****P < 0.0001; ***P < 0.001.

**FIGURE 4.**
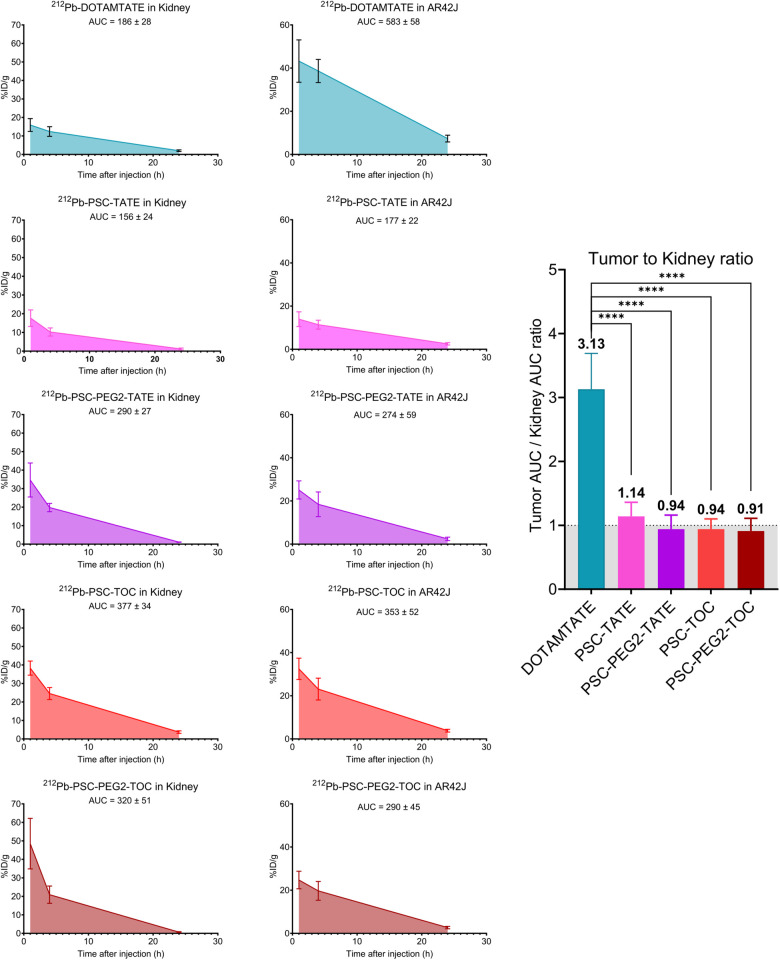
Tumor uptake AUC and ratios for ^212^Pb-radiolabeled SSTR2 analogs. Between 370 and 444 kBq of drug was administered, and organs were collected from 4–5 mice per time point: 1, 4, and 24 h after injection. Tissue uptake is expressed as %ID/g ± SD decay corrected to time of collection (*n* = 4–5). ****P < 0.0001.

In our study, we propose to use the integral of the time–activity curve, also known as the AUC, as a surrogate measure for estimating the absorbed dose. We calculated all SSTR2 analogs’ AUC for kidneys and tumors including tumor-to-kidney ratios (T/K AUC ratio) (Figs. 3 and 4). We observed distinct AUC patterns and consequently distinct T/K AUC ratios across the different compounds. Notably, [^212^Pb]Pb-DOTAMTATE demonstrated a significantly higher T/K AUC ratio of 3, indicating a favorable tumor uptake and retention relative to renal accumulation. The [^212^Pb]Pb-DOTAM-JR11 and [^212^Pb]Pb-PSC-TATE T/K AUC ratio was slightly above 1. [^212^Pb]Pb-PSC-PEG2-TATE, PSC-TOC, and PSC-PEG2-TOC presented a T/K AUC ratio of approximately 1 (Figs. 3 and 4).

## DISCUSSION

We performed a comprehensive study to evaluate the biodistribution of several ^212^Pb-radiolabeled SSTR2 analogs. These compounds, each with unique chemical properties, were evaluated side by side under identical conditions. This comparative approach allowed us to discern subtle differences in their distribution profiles, providing valuable insights into their expected dosimetry.

We found a higher initial uptake in the pancreas for both [^212^Pb]Pb-DOTAM-JR11 and [^212^Pb]Pb-DOTAMTATE likely due to the known SSTR2 expression in the pancreas (Fig. 2). However, we noted a faster washout from the pancreas than the tumor, consistent with previously published preclinical and clinical studies (19–21). The observed differential behavior may be attributed to variations in receptor metabolism between normal and neoplastic tissues. However, the precise underlying mechanism warrants further investigation. The accumulation in other (non–dose-limiting) healthy tissues appears distinctly cleaner for [^212^Pb]Pb-DOTATATE and all [^212^Pb]Pb-PSC SSTR2 analogs. Traditionally, agonist peptides such as octreotide have been used (22–26); however, antagonist peptides have demonstrated higher tumor uptake and longer tumor retention compared with their agonist counterparts (25,26). This is primarily because antagonists bind to a larger population of binding sites than agonists. For instance, in a comparative preclinical study, the SSTR2 antagonist ^177^Lu-OPS201 showed superior tumor uptake and longer tumor retention in SSTR2-expressing xenografts compared with the agonist [^177^Lu]Lu-DOTATATE (25). Consistently, in this study, [^212^Pb]Pb-DOTAM-JR11 has shown favorable tumor retention compared with other SSTR2-targeting analogs.

Historically, murine dosimetry has served as a valuable predictive model for the estimation of radiation doses to different organs. Despite the inherent differences between murine and human physiology, the dose-limiting tissue identification derived from these models has often shown a strong correlation with subsequent human studies. Therefore, we sought to determine the AUC as a surrogate measure for estimating the absorbed dose. This approach is based on the premise that the AUC (decay corrected to time of collection), which represents the total radioactivity over time, can provide a reliable estimate of the radiation dose absorbed by a specific tissue. Importantly, our investigation showed a superior T/K AUC ratio for [^212^Pb]Pb-DOTAMTATE (T/K AUC ratio of 3.13) over all other ^212^Pb-radiolabeled SSTR2 analogs (T/K AUC ratio of 0.91 to 1.4) (Fig. 3). Also, we observed that the addition of the PEG2 linker did not seem to have a positive impact on the compounds’ T/K AUC ratio, which appears to challenge the findings reported by Lee et al. (24). Our biodistribution data are consistent with theirs; however, in their study, the authors concluded that the PEG2 variant was advantageous compared with PSC-TOC based on biodistribution data. The T/K ratios were calculated using %ID/g values decay-corrected to the time of injection, which does not account for the reduced impact of ^212^Pb at 24 h due to its decay. In contrast, our study calculates the AUC and ratios on the basis of values decay-corrected to the time of collection, reflecting the significant contribution of ^212^Pb α-emission at 4 h compared with 24 h. Our data demonstrated T/K AUC ratios of 0.94 and 0.91 for PSC-TOC and PSC-PEG2-TOC, respectively, challenging the benefit of the PEG2 linker. Similarly, other studies reported a deterioration after addition of PEG linkers; for instance, [^68^Ga]Ga-DOTA-PEG4-TATE demonstrated instability in murine serum (21).

Additionally, we compared the chelation efficiency of DOTA as well as the 2 chelators dedicated to ^212^Pb chelation, namely DOTAM and PSC (Table 1). We demonstrated that an over 95% RCY of ^212^Pb at the specific activity of 444 MBq per 27.5 nmol happened in few minutes at room temperature for DOTAM, whereas DOTA required an additional 20-min incubation at room temperature. Despite its relatively rapid chelation kinetic, the DOTA chelator represents a suboptimal option for ^212^Pb radiolabeling because of its poor stability at low pH (14). The primary concern is its impact on toxicity rather than biodistribution, thus the conclusions regarding its distribution and potential absorbed dose remain valid. PSC, however, required approximately 30 min at room temperature and an additional 20 min at 37°C to reach an over 95% RCY. The PSC has distinctively required elevated temperature and longer incubation as it has been reported to preferentially be heated to approximately 80–85°C for a duration of up to 30 min (27–29). Therefore, we concluded that the DOTAM chelator presents a better complexation kinetic with ^212^Pb than do DOTA and PSC. All chelators, however, presented a RCY exceeding 95% 24 h after chelation with ^212^Pb.

Moreover, it is worth noting that our research has elucidated the origin of the previously observed discrepancy in quantification where administered activities were reported to be up to 3.7 MBq for ^212^Pb-radiolabeled compounds with no reported toxicity (16). Recent corrections to the quantification methodology have rectified this issue (30). Consequently, the described activities now align more accurately with the anticipated ranges for both efficacious and toxic doses for ^212^Pb radiopharmaceutical therapies in preclinical studies.

## CONCLUSION

The collected data suggest a faster chelation efficiency of the DOTAM and DOTA chelator over PSC. We discovered that minor modifications can significantly impact a compound’s pharmacokinetics. We also found that [^212^Pb]Pb-DOTAMTATE exhibits an advantageous dosimetry profile, which is essential for the management of SSTR2-positive NETs.

## DISCLOSURE

No potential conflict of interest relevant to this article was reported.

## Notes

KEY POINTS **QUESTION:** What are the comparative pharmacokinetic properties of [^212^Pb]Pb-DOTAMTATE and other somatostatin-targeting compounds in preclinical models? **PERTINENT FINDINGS:** The findings highlight the superior chelation efficiency and faster kinetics of DOTAM compared with those of PSC. It was discovered that [^212^Pb]Pb-DOTAMTATE has a superior T/K AUC ratio over other SSTR2-targeting peptides when radiolabeled with ^212^Pb, indicating favorable tumor retention and a more favorable dosimetry profile. **IMPLICATIONS FOR PATIENT CARE:** The results suggest that [^212^Pb]Pb-DOTAMTATE could potentially offer a more effective targeted α-therapy treatment option for SSTR2-positive NETs with a favorable tumor retention and dosimetry profile.
